# Curcumin inhibits human retinal pigment epithelial cell proliferation

**DOI:** 10.3892/ijmm.2014.1861

**Published:** 2014-07-23

**Authors:** YUN SUN, ZHI-PENG YOU

**Affiliations:** Department of Ophthalmology, The Second Affiliated Hospital of Nanchang University, Nanchang, Jiangxi 330006, P.R. China

**Keywords:** curcumin, proliferative vitreoretinopathy, retinal pigment epithelial cell, p53, p21^WAF1,CIP1^, proliferating cell nuclear antigen

## Abstract

Proliferative vitreoretinopathy (PVR) is a common cause of intraoperative failure following retinal reattachment surgery and is mediated in part through the migration, de-differentiation and proliferation of retinal pigment epithelial (RPE) cells. Given the cytotoxic effects of curcumin on epithelial and endothelial cells, in this study, we assessed the effects of curcumin on human RPE (hRPE) cell proliferation. WST-1 analysis revealed that curcumin significantly inhibited primary hRPE cell proliferation in a dose- and time-dependent manner (P<0.001) with the greatest inhibition observed at the dose of 15 μg/ml curcumin. Flow cytometric analysis indicated that the cytotoxic effects of curcumin on hRPE cell proliferation were mediated by cell cycle arrest at the G0/G1 phase and the induction of apoptosis (both P<0.001), which was confirmed by ultrastructural analysis using transmission electron microscopy. Furthermore, western blot analysis revealed that curcumin induced p53 and p21^WAF1/CIP1^ expression with a concomitant decrease in proliferating cell nuclear antigen protein levels (P<0.05). Curcumin effectively inhibited primary hRPE cell proliferation, which may be mediated by the p53 pathway. Further *in vivo* studies are required in order to fully explore the therapeutic potential of curcumin for PVR.

## Introduction

Retinal pigment epithelial (RPE) cells are a highly specific cell monolayer localized between the choroid and retina that transports nutrients from the choroid to photoreceptors, phagocytizes outer segments of the photoreceptor and maintains retinal adhesion and intraocular pressure balance through water and ion transport (1,2). The migration, de-differentiation and proliferation of RPE cells play important roles in the pathogenesis of proliferative vitreoretinopathy (PVR) (3–7), a common cause of intraoperative failure following retinal reattachment surgery, occurring in 5–10% of patients with rhegmatogenous retinal detachment (8). Specifically, the diffusion of RPE cells into the intravitreal cavity induces the formation of a retraction membrane on the retina, resulting in the vitreous retraction of fibrous tissues and retinal detachment (8–10). Currently, the only effective strategy for the treatment of hyperplastic fibrous tissues is surgical detachment; however, repeated surgeries may result in PVR (11).

The mechanisms underlying the migration and proliferation of RPE cells remain poorly understood; however, the role of growth factors and cytokines (3–7,12) in addition to factor Xa and thrombin (13) has been reported. The hyperplastic fibrous tissues of PVR are mainly composed of cells, including RPE cells, Müller glia, fibroblasts and macrophages, as well as an extracellular matrix (ECM) composed of collagen I and fibronectin (14,15). In addition, growth factor expression, including insulin-like growth factor-binding protein-6 (IGFBP-6) (16) and vascular endothelial growth factor A (VEGF-A) (17), has been observed in the vitreous of PVR patients. The inhibition of the migration and proliferation of these cells may be helpful in preventing the recurrence of retinal detachment (18,19). Although controversial, some clinicians have used the intravitreal or systemic injection of steroids to inhibit cell proliferation with poor efficacy (20,21). In addition, although the inhibition of RPE cell proliferation *in vitro* has been achieved using tranilast (22), genistein (23), ciprofloxacin (24), vitamin C (25), vitamin E (26), minoxidil (27), hypericin (28), *cis*-hydroxyproline (29), retinoic acid (25), *cis*-vitamin A acid, aclacinomycin A (30), daunorubicin (31) and N,N-dimethyl doxorubicin (32), many of these drugs have side effects, which significantly limit their wide application in clinical practice.

In recent years, studies have demonstrated that curcumin, derived from the rhizome of *Curcuma longa*, may inhibit the proliferation of endothelial and epithelial cells; however, few studies have assessed its effects on RPE cells (33,34). Therefore, this study aimed to examine the hypothesis that curcumin inhibits RPE cell proliferation. In addition, the possible mechanisms underlying the effects of curcumin were analyzed by determining the expression of pro-apoptotic factors (e.g., p53 and p21^WAF1/CIP1^) and proliferating cell nuclear antigen (PCNA).

## Materials and methods

### In vitro culture of human RPE (hRPE) cells

Primary RPE cells were obtained from ScienCell Research Laboratories (Carlsbad, CA, USA) which characterized them by immunofluorescence staining of cytokeratin-18, -19 and fibronectin. Cells infected with HIV-1, HCV, mycoplasma, bacteria, yeast and fungi were excluded. The cells were maintained in Dulbecco’s modified Eagle’s medium (DMEM)/F12 (Invitrogen, Carlsbad, CA, USA) containing 10% fetal bovine serum (FBS), 100 U/ml penicillin and 100 mg/ml streptomycin (all from Invitrogen) at 37°C in a humidified environment with 5% CO_2_. The cells were passaged at a ratio of 1:2 when they reached 80% confluence after 2–3 days in culture.

### Cell proliferation assay

The WST-1 kit (Roche, Indianapolis, IN, USA) was used to detect RPE cell proliferation and viability following the manufacturer’s instructions. Briefly, the hRPE cells at passage 5 who had achieved a satisfactory growth were used to prepare a single-cell suspension. The cells were seeded onto 96-well plates (5,000 cells/well in 100 μl) and incubated at 37°C with 5% CO_2_. The cells were treated with 5, 10, 15, 20 μg/ml curcumin (dissolved in DMSO; Sigma-Aldrich, St. Louis, MO, USA) or DMSO. After 24, 48 and 72 h, 10 μl of WST-1 solution were added to each well, followed by incubation for 1–4 h. The optical density (OD) was measured at 450 nm to determine the optimal concentration of the curcumin-mediated inhibition of proliferation.

### Detection of apoptosis

The Annexin V-FIT apoptosis detection kit I (BD Pharmingen, San Jose, CA, USA) was used to detect curcumin-induced apoptosis. In brief, the hRPE cells were treated with 15 μg/ml curcumin or DMSO (control) for 48 h, digested with EDTA-free trypsin (Invitrogen) and then washed twice with phosphate-buffered saline (PBS). Approximately 1–5×10^5^ cells were collected and re-suspended in 500 μl of binding buffer, 2 μl of Annexin V-FITC and 5 μl of propidium iodide (PI), followed by incubation in the dark at room temperature for 5 min. Flow cytometry (using a flow cytometer; Beckman Coulter, Brea, CA, USA) was performed at an excitation of 488 nm and an emission of 530 nm to detect the apoptotic cells. Cells positive for Annexin V-FITC, but negative for PI were considered apoptotic; those positive for both Annexin V-FITC and PI were considered necrotic.

### Cell cycle analysis

The hRPE cells were treated with 15 μg/ml curcumin or DMSO (control) for 48 h, digested with EDTA-free trypsin (Invitrogen), washed twice with PBS and harvested by centrifugation. Following fixation in 70% ethanol at 4°C overnight, the cells were washed with 5 ml of PBS and incubated with 500 μl of PBS containing 100 μg/ml PI (Sigma-Aldrich), 100 μg/ml RNase A (Sigma-Aldrich) and 0.2% Triton X-100, followed by incubation at 4°C in the dark for 30 min and flow cytometry.

### Transmission electron microscopy

Following treatment with 15 μg/ml curcumin for 24, 48 and 72 h, the hRPE cells were harvested and seeded onto coverslips, which were washed twice with PBS and fixed in 2.5% glutaraldehyde at room temperature for 1 h and then in 2% osmic acid. Following washing in 0.1 M PBS followed by distilled water, the cells were dehydrated in a graded ethanol series. Following treatment with Poly(diallyl phthalate) (PDAP; Sigma-Aldrich) and *in situ* embedding, ultra-thin sections were obtained, which were stained with 2% uranyl acetate and lead citrate. These sections were observed under a transmission electron microscope (JEM-1230; Joel, Tokyo, Japan).

### Western blot analysis

Following treatment with 15 μg/ml curcumin for 24, 48 and 72 h, the hRPE cells were harvested and lysed in RIPA lysis buffer (50 mM Tris, pH 7.4; 150 mM NaCl; 1% Triton X-100; 1% sodium deoxycholate; 0.1% SDS; sodium orthovanadate; sodium fluoride; EDTA; leupeptin), and the protein concentration was determined using the BCA method. Proteins (50 μg) were separated with 12% SDS-PAGE and transferred onto PVDF membranes (Millipore, Billerica, MA, USA), which were incubated in blocking buffer (TBS, 0.1% Tween-20, 2% BSA) at room temperature for 1 h. After the membranes were washed 3 times in TBS (5 min/wash), they were incubated with the following primary antibodies at room temperature for 1.5 h: mouse anti-human p21^WAF1/CIP1^ monoclonal antibody, mouse anti-human p53 monoclonal antibody, mouse anti-human PCNA monoclonal antibody (all from Santa Cruz Biotechnology, Inc., Dallas, TX, USA) and glyceraldehyde 3-phosphate dehydrogenase (GAPDH) monoclonal antibody (Cell Signaling Technology, Danvers, MA, USA). After washing in TBS, the membranes were incubated with HRP-conjugated secondary antibodies (1:2,000; Santa Cruz Biotechnology, Inc.) at room temperature for 1 h followed by chemiluminescence detection with an ECL GST Western Blotting Detection kit (Santa Cruz Biotechnology, Inc.). Quantity One software (Bio-Rad Laboratories, Hercules, CA, USA) was used to determine the OD of the bands, and GAPDH served as an internal reference.

### Statistical analysis

Continuous data are presented as the means ± standard deviation (SD). Curcumin-induced hRPE inhibition by dose at a given time or among different time points at a given dose was compared by one-way analysis of variance (ANOVA) with pair-wise post-hoc tests using Bonferroni correction. The apoptotic rate, cell necrotic rate, cell cycle and protein expression between the control and curcumin-treated groups were compared by an independent t-test. All data analyses were performed using SPSS statistical software (version 17.0; SPSS Inc., Chicago, IL, USA). A two-tailed P-value <0.05 indicated a statistically significant difference.

## Results

### Inhibitory effects of curcumin on hRPE cell proliferation in vitro

The curcumin-mediated inhibition of hRPE cell proliferation was assessed following treatment with different doses of curcumin after 24, 48 and 72 h. A dose-dependent inhibition in hRPE cell proliferation was observed at each time point analyzed (P<0.001) (Table I). Moreover, the inhibitory effects of curcumin significantly increased with the increase in the treatment duration at each curcumin concentration (P<0.001). For both the 48- and 72-h time points, the proliferation inhibition rate of the cells treated with 15 μg/ml curcumin was significantly higher than that of the cells treated with 5 and 10 μg/ml curcumin at the same time points (all P<0.05). The proliferation inhibition rate of the cells treated with 20 μg/ml curcumin was significantly higher than that of the cells treated with 5 and 10 μg/ml curcumin at all 3 time points; however, it did not differ significantly from that of the cells treated with 15 μg/ml curcumin (Table I). Therefore, the dose of 15 μg/ml curcumin was selected for the subsequent experiments.

### Effects of curcumin on hRPE cell apoptosis and necrosis

**Figure 1:**
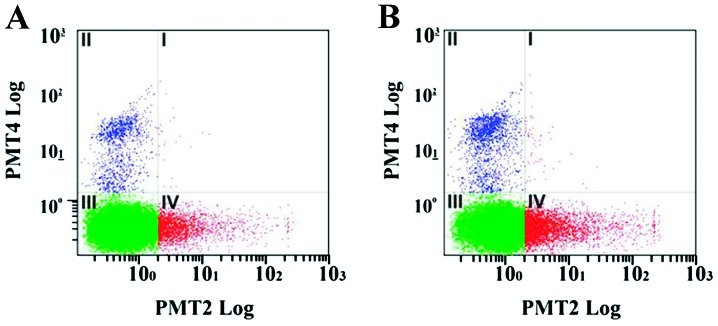
Effect of curcumin on the apoptosis and necrosis of human retinal pigment epithelial (hRPE) cells. Apoptotic and necrotic rates in (A) control and (B) curcumin-treated hRPE cells (15 μg/ml curcumin for 48 h). First quadrant, propidium iodide (PI) staining (necrotic cells); second quadrant, PI and Annexin V-FITC double staining (middle to late phase of apoptosis); forth quadrant: Annexin V-FITC staining (early phase of apoptosis).

The hRPE cells were treated with 15 μg/ml curcumin for 48 h after which the proportion of apoptotic and necrotic cells was determined; representative data are shown in Fig. 1. As shown in Table II, the curcumin-treated group had a significantly greater proportion of cells in the early apoptotic phase (13.37 vs. 7.03%; P=0.001), resulting in a significantly higher overall apoptotic rate (P=0.001) compared to the control group. However, no significant difference was observed in the proportion of necrotic cells between the 2 groups (Table II).

**Figure 2:**
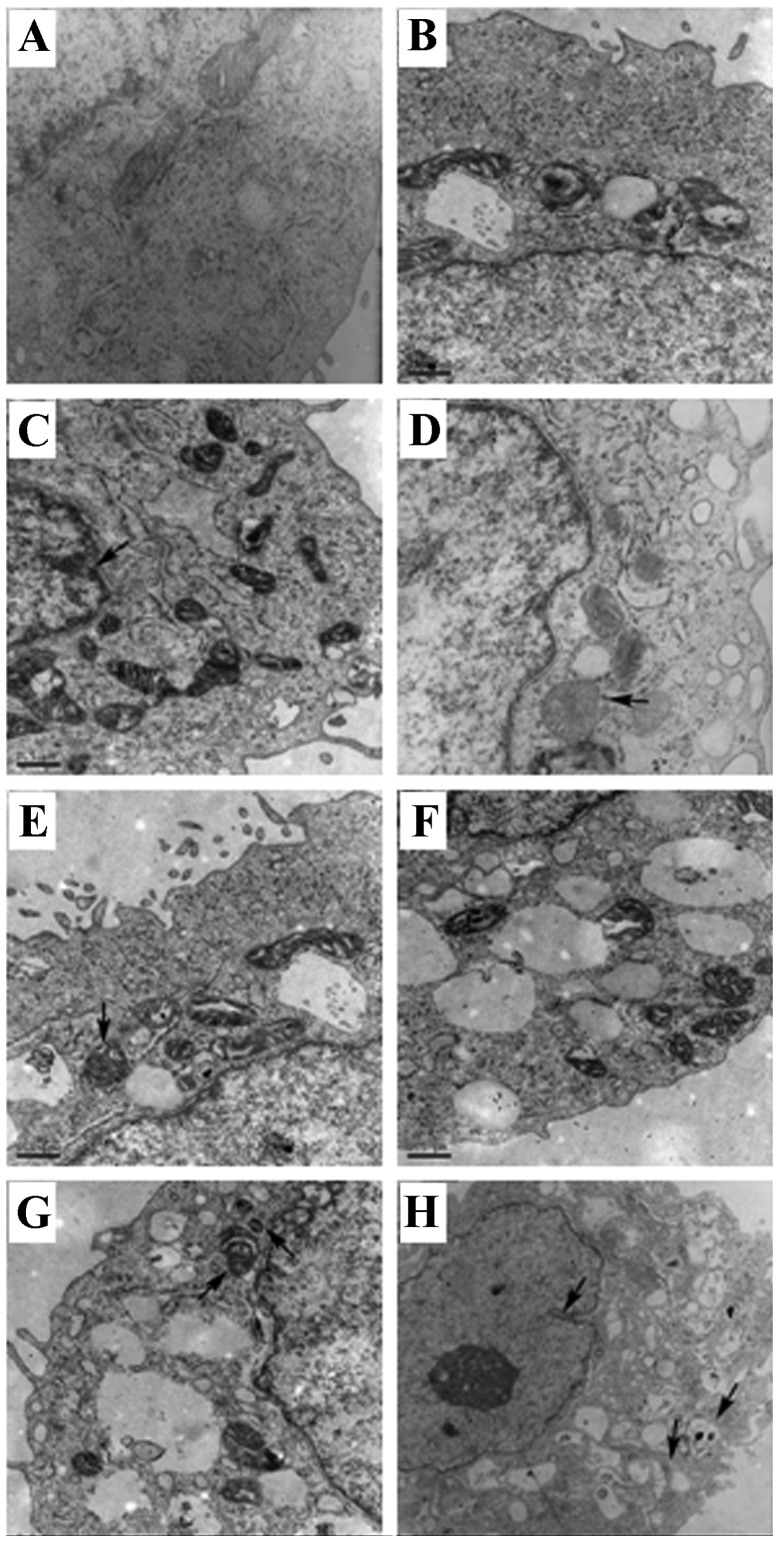
Ultrastructural analysis of human retinal pigment epithelial (hRPE) cells following treatment with curcumin. (A) hRPE cells in the normal control group (TEM ×20,000; scale bar, 0.5 μm). (B and C) hRPE cells in the early phase of apoptosis (TEM ×20,000; scale bar, 0.5 μm). The arrow in panel C indicates chromatin margination. (D–G) hRPE cells in the middle-to-late phase of apoptosis (TEM ×20,000; scale bar, 0.5 μm). The arrows in panels D and E indicate mitochondrial swelling. The arrow on the left side of panel G indicates mitochondrial swelling, and the arrow on the right side of panel G indicates a lysosome. (H) hRPE cells in the late phase of apoptosis (TEM ×10,000, scale bar, 1 μm). The arrow on the left side indicates nuclear membrane shrinkage, and the arrows on the right side indicate damaged, vesicular organelles.

A subsequent analysis of the effects of curcumin on the hRPE cell ultrastructure after 48 h was undertaken using transmission electron microscopy (Fig. 2). While the absence of apoptosis-induced changes in the cellular ultrastructure was noted in the normal control cells (Fig. 2A), changes associated with the different phases of apoptosis were observed in the curcumin-treated cells (Fig. 2B–H). Specifically, in early apoptosis, chromatin margination was detected along with a slight increase in the number of mitochondria, mitochondrial swelling and lysosomes (Fig. 2B and C). In middle-to-late apoptosis, the hRPE cells exhibited nuclear membrane shrinkage, mitochondrial swelling (Fig. D–F), as well as a large amount of lysosomes (Fig. 2F). In the late phase of apoptosis, characteristics similar to those of necrotic cells (i.e., rupture of the cell membrane, nuclear membrane shrinkage, injured organelles and cytoplasmic vacuoles) were observed (Fig. 2H).

### Effects of curcumin on hRPE cell cycle progression

**Figure 3:**
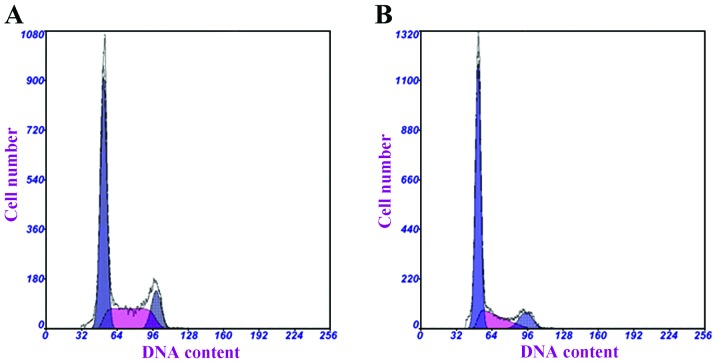
Effect of curcumin on the cell cycle progression of human retinal pigment epithelial (hRPE) cells. Cell cycle analysis of (A) control and (B) curcumin-treated hRPE cells (15 μg/ml curcumin for 48 h). Dark blue color represents the G0/G1 phase, purple represents the S phase and light blue represents the G2/M phase.

The cell cycle progression of hRPE cells was then determined following treatment with 15 μg/ml curcumin for 48 h (Fig. 3). The proportion of hRPE cells in the G0/G1 phase was significantly higher in the curcumin-treated group than in the control group (67.73 vs. 57.17%; P<0.001) (Table III). A concomitant decrease in the proportion of curcumin-treated hRPE cells in the S phase was observed (P=0.010).

### Effects of curcumin on p21^WAF1/CIP1^, p53 and PCNA protein expression in hRPE cells

**Figure 4:**
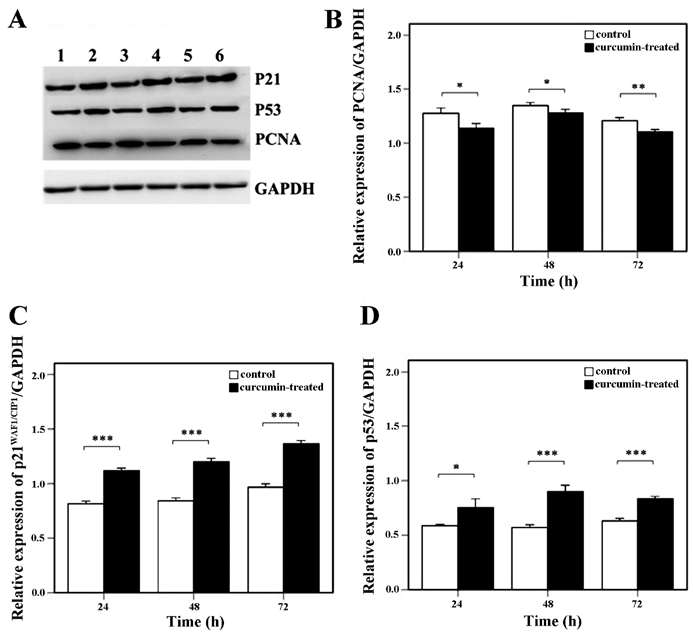
Effects of curcumin on cell cycle-related protein levels. (A) A representative western blot is shown. Lane 1, control at 24 h; lane 2, curcumin treatment for 24 h; lane 3, control at 48 h; lane 4, curcumin treatment for 48 h; lane 5, control at 72 h; and lane 6, curcumin treatment for 72 h. The relative protein expression of (B) proliferating cell nuclear antigen (PCNA), (C) p21^WAF1/CIP1^ and (D) p53 was determined in cultured hRPE cells. Data represent the means ± standard deviation (SD) of 3 independent experiments for each time point. *P<0.05, ^**^P<0.01 and ^***^P<0.001: significantly different from the control group.

The hRPE cells were treated with 15 μg/ml curcumin for 24, 48 and 72 h after which western blot analysis was undertaken; a representative blot is shown in Fig. 4A. The relative PCNA expression was significantly decreased in the curcumin-treated group as compared to the control group at all time points (P<0.05) (Fig. 4B); however, the relative expression of p21^WAF1/CIP1^ (Fig. 4C) and p53 (Fig. 4D) was significantly elevated in response to curcumin treatment (all P<0.05).

## Discussion

Given the anti-proliferative effects of curcumin for epithelial and endothelial cells, its effects on hRPE cell proliferation were determined in the present study. Similarly, curcumin inhibited hRPE cell proliferation in a dose-dependent manner. The anti-proliferative response by curcumin was mediated in part by increased apoptosis, as well as G0/G1 cell cycle arrest. No induction of necrosis was observed. Furthermore, curcumin increased p21 and p53 protein expression, while decreasing PCNA protein levels.

Curcumin is a diarylheptanoid extracted from the rhizome of *Curcuma longa*. *In vitro* studies have demonstrated the antioxidant (35), anti-inflammatory (36), anti-angiogenic (37) and anti-proliferative (38) effects of curcumin in a variety of cells. In the present study, curcumin significantly inhibited the proliferation of hRPE cells *in vitro* in a dose- and time-dependent manner. This is consistent with the cytotoxic effects of curcumin in hRPE cells reported by Hollborn *et al* (33).

As the WST-1 assay only indirectly reflects cell viability without directly assessing cell proliferation or cell death, we also assessed the effects of curcumin on apoptosis and necrosis. Although no significant induction of necrosis was observed, treatment with 15 μg/ml curcumin for 48 h significantly increased the apoptotic rate, which was confirmed by transmission electron microscopy that detected ultrastructural changes consistent with the early and middle-to-late phases of apoptosis. Hollborn *et al* (33) reported increased hRPE cell necrosis in response to >10 μM curcumin. Differences in the concentration of curcumin used (>10 μM vs. 15 μg/ml), as well as the duration of culture (6 and 24 vs. 48 h) may account for the discrepant results with respect to necrosis.

In the present study, curcumin increased the expression of p53, which plays a role in the mitochondrial apoptotic pathway, as well as the expression of its downstream factor, p21^WAF1/CIP1^. This result is consistent with that of a previous study that reported the curcumin-induced apoptosis of retinal vascular endothelial cells (39). In hRPE cells and ARPE19 cells, curcumin has been shown to induce apoptosis through the mitochondrial apoptotic pathway (40). These results suggest that curcumin increases mitochondrial outer membrane permeability (MOMP), leading to the release of p53, which in turn elevates MOMP and thereby initiates apoptosis. Given the importance of the p53 pathway and its negative regulator, murine double minute 2 (MDM2), in PVR pathogenesis (41), further studies are required to assess this possibility in detail. Furthermore, curcumin-induced p53 and p21^WAF1/CIP1^ expression was detected at the same time point, suggesting that p53 initiates the expression of p21^WAF1/CIP1^. In a previous study, in hRPE cells treated with H_2_O_2_ for 5 min, an increased p21^WAF1/CIP1^ expression ensued that of p53 (42). Therefore, we hypothesized that treatment with curcumin may induce the release of p53 from the mitochondria at extremely early time points; however, prolonged curcumin treatment may induce the synthesis of p53 and p21^WAF1/CIP1^. As p53 suppresses VEGF, a growth factor relevant to PVR pathogenesis (17) and negatively regulated by curcumin (33), further studies are required to assess the expression of additional p53 downstream factors in response to curcumin, apart from p53.

In addition to inducing apoptosis, both p53 and p21^WAF1/CIP1^ mediate cell cycle arrest. p53 arrests cells in the G1 phase to impair DNA repair or induce apoptosis, and p21^WAF1/CIP1^ interacts with cyclin-dependent kinase (CDK)2 to arrest cells in the G1 phase, inhibiting DNA replication and mitosis (43–45). Adenovirus-mediated p21^WAF1/CIP1^ overexpression in retinal vascular endothelial cells inhibits their proliferation and tube formation (46). In the present study, curcumin induced G0/G1 phase arrest in the hRPE cells, which is consistent with that observed in human umbilical vein endothelial cells (HUVECs) (47). Moreover, the proportion of hRPE cells in the S phase was reduced with curcumin treatment, while the number of cells in the G2/M phase remained unaltered. These results were confirmed by evaluating the expression of PCNA, an important marker of cell proliferation involved in DNA synthesis, repair, regulation of the cell cycle, chromosomal rearrangement and DNA methylation. As a co-factor of DNA polymerase δ, PCNA participates in DNA synthesis and replication and regulates entry into the S phase; its expression is increased in the G1 phase, peaking in the S phase (48,49). In the present study, PCNA levels decreased in the hRPE cells treated with curcumin. This downregulation may be attributed to its increased interaction with p21^WAF1/CIP1^, which inhibits CDK activity, blocks retinoblastoma protein (Rb) phosphorylation and arrests cells before the S phase (50). Further studies are required to assess the mechanisms and factors through which curcumin inhibits cell cycle progression in greater detail, including assessing Krüppel-like factor 5 (KLF5), an inhibitor of aberrant cell cycle progression in epithelial cells which functions in part through the regulation of p21^WAF1/CIP1^ (51).

In addition to hRPE cell proliferation, hyperplastic fibrous tissues of PVR contain ECM components, including collagen I and sometimes collagen II (14). Further studies are required to determine the effects of curcumin on ECM components in PVR.

In conclusion, taken together, our data demonstrate that curcumin effectively inhibits the proliferation of primary hRPE cells in a time- and dose-dependent manner. The cytotoxic effects of curcumin are attributed to reduced cell cycle progression and the induction of apoptosis, which may be related to the p53 and p21^WAF1/CIP1^ upregulation and the decrease in PCNA expression. We hypothesize that the p53 signaling pathway is involved in the anti-proliferative effects of curcumin on hRPE cells. Further *in vivo* studies are required to fully explore the therapeutic potential of curcumin for PVR.

## Figures and Tables

**Table I:** The inhibitory effects of curcumin on the proliferation of cultured human retinal pigment epithelial (hRPE) cells.

	Curcumin concentration (μg/ml)				
		
Time (h)	5	10	15	20	P-value^a^
24	5.07±2.66	11.53±2.58	15.85±2.71^c^	21.32±1.67^c,d^	<0.001
48	11.24±0.44^e^	15.44±2.52	21.41±2.55^c,d^	26.38±1.09^c,d,e^	<0.001
72	18.85±1.60^e,f^	29.69±0.75^c,e,f^	39.96±3.88^c–f^	40.10±2.11^c–f^	<0.001
P-value^b^	<0.001	<0.001	<0.001	<0.001	

Inhibition rates (%) are presented as the means ± SD. N=3 for each dose at each time point. ^a^Overall test for the difference among groups by dose, determined by ANOVA. ^b^Overall test for the difference among groups over time, determined by ANOVA. Significantly different from the ^c^5, ^d^10 μg/ml group, ^e^24 and ^f^48 h group as determined by Bonferroni correction, P<0.05. SD, standard deviation; ANOVA, analysis of variance.

**Table II:** The effects of curcumin on the apoptosis and necrosis of cultured human retinal pigment epithelial (hRPE) cells after 48 h.

	Control group	Curcumin-treated group	P-value^a^
Apoptotic rate (%), early phase	7.03±0.37	13.37±1.26	0.001
Apoptotic rate (%), mid-late phase	0.09±0.00	0.16±0.09	0.307
Apoptotic rate (%), total	7.12±0.37	13.53±1.18	0.001
Cell necrosis rate (%)	4.40±0.18	5.19±0.75	0.154

Data are presented as the means ± SD; N=3 for each group.

a Determined by independent t-test.

SD, standard deviation.

**Table III:** The effects of curcumin on the cell cycle progression of cultured human retinal pigment epithelial (hRPE) cells after 48 h.

Cell cycle phase	Control group	Curcumin-treated group	P-value^a^
G0/G1 (%)	57.17±1.17	67.73±1.10	<0.001
S (%)	32.23±3.47	21.47±2.03	0.010
G2/M (%)	10.64±3.41	10.83±1.57	0.935

Data are presented as the means ± SD; N=3 for each group.

a Determined by independent t-test.

SD, standard deviation.

## Abbreviations

ANOVA: analysis of variance
ECM: extracellular matrix
FBS: fetal bovine serum
OD: optical density
PCNA: proliferating cell nuclear antigen
PVR: proliferative vitreoretinopathy
RPE cells: retinal pigment epithelial cells
SD: standard deviation
